# HLA-Arena: A Customizable Environment for the Structural Modeling and Analysis of Peptide-HLA Complexes for Cancer Immunotherapy

**DOI:** 10.1200/CCI.19.00123

**Published:** 2020-07-15

**Authors:** Dinler A. Antunes, Jayvee R. Abella, Sarah Hall-Swan, Didier Devaurs, Anja Conev, Mark Moll, Gregory Lizée, Lydia E. Kavraki

**Affiliations:** ^1^ Department of Computer Science, Rice University, Houston, TX; ^2^ Université Grenoble Alpes, Inria, Grenoble, France; ^3^ Department of Melanoma Medical Oncology–Research, The University of Texas MD Anderson Cancer Center, Houston, TX

## Abstract

**PURPOSE:**

HLA protein receptors play a key role in cellular immunity. They bind intracellular peptides and display them for recognition by T-cell lymphocytes. Because T-cell activation is partially driven by structural features of these peptide-HLA complexes, their structural modeling and analysis are becoming central components of cancer immunotherapy projects. Unfortunately, this kind of analysis is limited by the small number of experimentally determined structures of peptide-HLA complexes. Overcoming this limitation requires developing novel computational methods to model and analyze peptide-HLA structures.

**METHODS:**

Here we describe a new platform for the structural modeling and analysis of peptide-HLA complexes, called HLA-Arena, which we have implemented using Jupyter Notebook and Docker. It is a customizable environment that facilitates the use of computational tools, such as APE-Gen and DINC, which we have previously applied to peptide-HLA complexes. By integrating other commonly used tools, such as MODELLER and MHCflurry, this environment includes support for diverse tasks in structural modeling, analysis, and visualization.

**RESULTS:**

To illustrate the capabilities of HLA-Arena, we describe 3 example workflows applied to peptide-HLA complexes. Leveraging the strengths of our tools, DINC and APE-Gen, the first 2 workflows show how to perform geometry prediction for peptide-HLA complexes and structure-based binding prediction, respectively. The third workflow presents an example of large-scale virtual screening of peptides for multiple HLA alleles.

**CONCLUSION:**

These workflows illustrate the potential benefits of HLA-Arena for the structural modeling and analysis of peptide-HLA complexes. Because HLA-Arena can easily be integrated within larger computational pipelines, we expect its potential impact to vastly increase. For instance, it could be used to conduct structural analyses for personalized cancer immunotherapy, neoantigen discovery, or vaccine development.

## INTRODUCTION

Immunotherapy treatments are now at the forefront of methods used for cancer therapy. These treatments aim at harvesting a patient’s own immunologic defenses to identify and eliminate cancer cells.^1^ Many of these immunotherapy treatments involve class I HLA protein receptors. HLA receptors bind peptides produced by the cleavage of intracellular proteins, which is a continuous process present in almost every cell. The resulting peptide-HLA (pHLA) complexes are then exposed at the surface of cells. Also present in cancer cells, this mechanism allows circulating T-cell lymphocytes to recognize tumor-associated peptides, thus triggering T-cell activation, tumor elimination, and immunologic memory against the tumor.^1,2^

**CONTEXT**

**Key Objective**
Enabling large-scale structural modeling and analysis of peptide-HLA complexes for cancer immunotherapy applications.
**Knowledge Generated**
We created a customizable environment, called HLA-Arena, with user-friendly computational workflows that allow for varied structure-based analyses of peptide-HLA complexes. To illustrate this, we show how researchers can use HLA-Arena to perform geometry prediction of peptide binding modes, peptide binding energy prediction, and structure-based virtual screening of tumor-derived peptides, for any classic class I HLA of interest.
**Relevance**
HLA-Arena can be integrated in computational pipelines to support basic cancer research or to help inform physicians in preclinical settings. It can be used to perform structure-based selection of peptides for T cell–based immunotherapy, neoantigen discovery, and vaccine development.

It has been shown that immunologic outcomes are partially driven by structural features of pHLA complexes.^2-4^ Therefore, the structural modeling and analysis of these complexes are becoming essential to ensure the efficacy and safety of immunotherapy treatments.^2^ However, pHLA structural features are affected by the genetic variability of both patients and tumors.^2,5^ First, the set of peptides available for presentation reflects the patient’s genetic background and cancer-specific alterations.^2,5^ Second, each individual has up to 6 class I HLA alleles,^6^ among the nearly 19,000 alleles in the human population.^7^ Each allele encodes for a receptor with specific characteristics, which will display a different pool of peptides. Therefore, the structural modeling and analysis of pHLA complexes for cancer immunotherapy require fast and customizable methods that can handle patient-specific data.

Unfortunately, the cost and time requirements of gold-standard experimental techniques in structural biology prevent their use in personalized medicine. In addition, few structures of pHLA complexes have been determined experimentally. Therefore, researchers have turned toward computational methods for the structural modeling of pHLA complexes. However, the length and flexibility of displayed peptides represent major challenges for traditional methods.^5^ As an alternative, in previous work, we have developed several computational tools for the accurate and efficient modeling of pHLA complexes. For example, we have described a fast method, called APE-Gen, to generate ensembles of peptide conformations bound to a given HLA receptor.^8^ We have also developed a meta-docking approach, called DINC, which allows prediction of binding modes of pHLA complexes.^9,10^

In this report, we present a higher-level platform, called HLA-Arena, that allows the carrying out of sophisticated structural modeling and analysis of pHLA complexes. Instead of having to deal with several computational tools, HLA-Arena provides researchers with a single customizable environment that fully integrates the tools we have developed, as well as other commonly used software. HLA-Arena simplifies the interactions with these tools by leveraging the capabilities of Jupyter Notebook and Docker. It allows users to perform various workflows, each involving a specific combination of tools and steps within a coherent scenario. In addition to APE-Gen and DINC, HLA-Arena currently integrates MODELLER^11^ for homology modeling, MHCflurry^12^ for binding affinity prediction, and NGL Viewer^13^ for structure visualization, among others.

Here, we present 3 example workflows illustrating the capabilities of HLA-Arena. The first relies on DINC to predict the binding modes of 2 known peptides with their corresponding HLA receptors (ie, geometry prediction). The second relies on APE-Gen to assess differences in binding between peptides restricted to a given HLA receptor, based on generated binding mode ensembles (ie, binding prediction). The third aims at performing structure-based virtual screening, which requires speed and scalability. Using real immunopeptidomic data and a fictitious diplotype (ie, 6 classic class I HLA alleles), we show how MHCflurry and APE-Gen can complement each other to select target peptides for a hypothetic immunotherapy treatment.

## METHODS

### Computational Approaches for pHLA Binding Mode Prediction

Despite their huge sequence diversity, HLA receptors feature conserved secondary and tertiary structures, as illustrated by available data.^14-16^ Such conserved folding makes HLA modeling an easy task with tools leveraging homology modeling.^8,17,18^ In contrast, predicting the binding modes of peptides to HLA receptors is much harder because of the size and flexibility of these peptides. As recently reviewed, strategies used to overcome this challenge include constrained backbone prediction, constrained termini prediction, and incremental prediction.^5^

**FIG 1.**
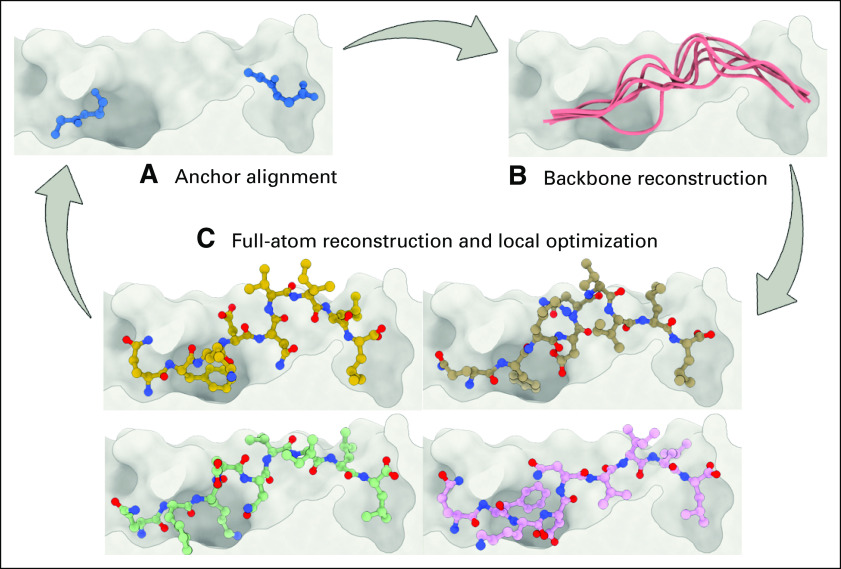
Generating binding mode ensembles with APE-Gen. (A) Templates of backbone termini are used to position the anchor residues of a peptide in the binding site. (B) The random coordinate descent loop closure tool^19^ is used to generate an ensemble of backbone conformations for this peptide. (C) Full-atom reconstruction of peptide side chains and local optimization of the resulting complex are performed for each sampled backbone. The highest-quality binding mode can be selected to be used as a template for the next round of the iterative process.

**FIG 2.**
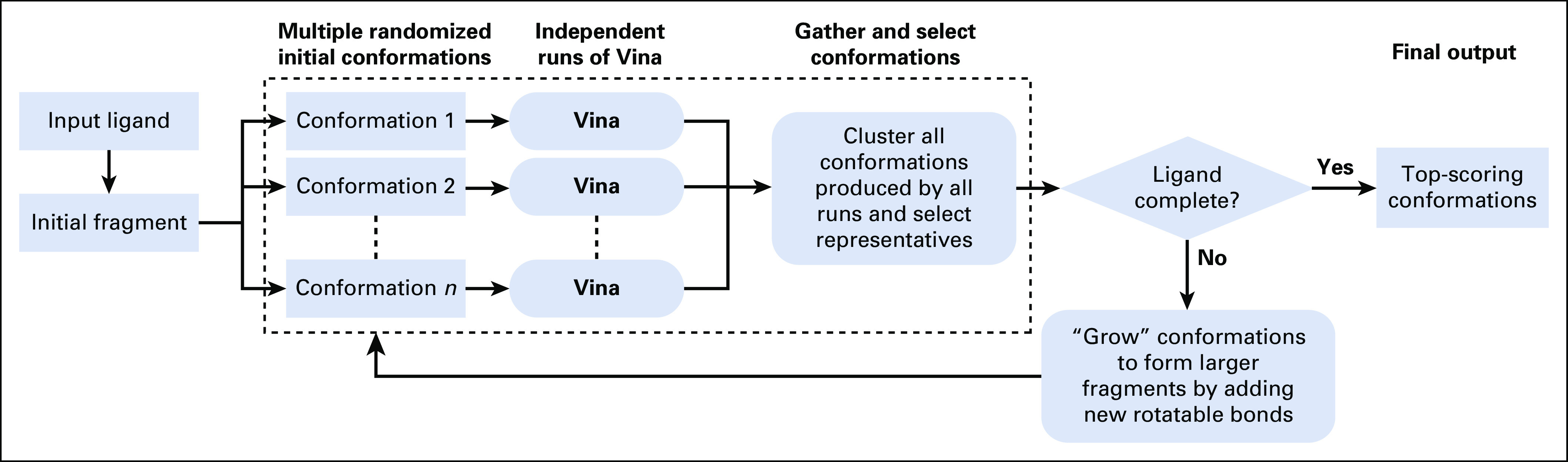
Workflow of DINC parallel and incremental meta-docking approach. DINC starts by selecting a small fragment of the input ligand, with only *k* flexible bonds. Multiple conformations are created by randomly sampling different values for the dihedral angles of this fragment. These *n* conformations are then used as input for multiple independent runs of a docking tool (in this example, Vina^20^), which are executed in parallel by different threads. From all the binding modes produced by these parallel runs, the *n* best modes are selected for expansion; they are grown by adding several atoms and bonds from the input ligand. These larger fragments are then docked independently, in parallel, while keeping the number of flexible bonds equal to *k*. This process is repeated until the entire input ligand has been incrementally reconstructed and is docked in the binding site of the receptor.

In recent years, we have implemented 2 computational approaches for pHLA binding mode prediction using these strategies. The first, called APE-Gen (anchored peptide-MHC ensemble generator), can quickly produce an ensemble of binding modes for a pHLA complex, using termini templates to position the peptide in the HLA binding cleft (Fig 1; Appendix).^8^ The second, called DINC, can incrementally dock a peptide in the binding site and does not require any template (Fig 2; Appendix).^9,10,21^ Each approach has different strengths and limitations and can therefore suit various user needs, depending on the task at hand. For instance, its speed makes APE-Gen better suited for large-scale modeling and structure-based virtual screening. In contrast, because it does not rely on templates, DINC’s predictions can be more general and account for unusual binding modes, thus making it more suited for geometry prediction.^9,22^ Both APE-Gen and DINC have been validated in previous publications.^8,10,23^ In this report, we present a unified environment that facilitates the use of APE-Gen, DINC, and other tools for various research applications.

### HLA-Arena: Structural Modeling and Analysis of pHLA Complexes

**FIG 3.**
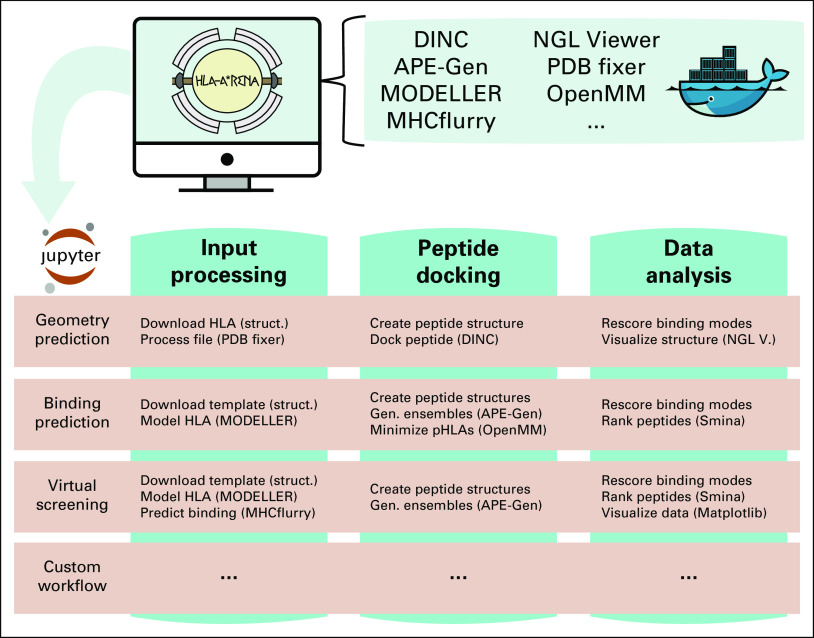
HLA-Arena leverages Docker and Jupyter Notebook, offering a customizable environment to build and execute various workflows for the structural modeling and analysis of peptide-HLA (pHLA) complexes. Three proposed workflows are depicted here: (1) geometry prediction of pHLA binding modes, (2) structure-based prediction of binding energy, and (3) virtual screening of tumor-derived peptides. In the geometry prediction workflow, after obtaining the structure of an HLA receptor, a peptide of interest is docked in its binding site by DINC, and all generated (Gen) binding modes are scored with several scoring functions. In the binding prediction workflow, after modeling a given HLA structure (struct.), ensembles of binding modes are generated with APE-Gen (and optionally minimized with OpenMM^24^) for various peptides, and these binding modes are scored to rank the peptides with Smina.^25^ In the virtual screening workflow, after filtering peptides with MHCflurry,^12^ ensembles of binding modes are generated with APE-Gen for the selected peptides, and the top-scoring binding modes are used to rank these peptides with Smina, in terms of binding affinity to an HLA receptor or set of receptors. Note that these workflows can be modified, and new workflows can be created by users. In each application, different types of data analysis can be used to guide the selection of the best pHLAs before experimental validation. The screen icon was modified from Flaticon.^26^ PDB, Protein Data Bank; V, Viewer.

Using Jupyter Notebook and Docker, we have created a customizable environment, called HLA-Arena, that enables researchers to easily model any class I pHLA complex of interest and perform varied structural analyses (Fig 3). HLA-Arena includes different workflows, defined as separate notebooks, which consist of the following main stages:

#### Input processing.

Available structures of HLA receptors are obtained from the Protein Data Bank (PDB)^27^ to be used as such or as templates. Unavailable HLA structures are modeled with MODELLER,^11^ using an HLA sequence and the structure of a similar HLA receptor as template, if these are provided by the user. Alternatively, users can just provide an allele name (eg, HLA-A*24:02); HLA-Arena will then fetch the proper sequence from IMGT/HLA,^7^ and a reasonable template (based on the HLA supertype^28^ classification) from the PDB. In addition, binding affinity of peptides can be estimated with MHCflurry^12^ to select the most relevant ones. Minimal example: HLA_allele = arena.model_hla(‘HLA-A*24:02’)

#### Peptide docking.

Structures of pHLA complexes are modeled with APE-Gen and/or DINC, which only require the sequence of the target peptide(s) and the HLA structure(s) obtained previously. Modeled structures can also be minimized with a force field using OpenMM.^24^ Minimal example: structure = arena.dock(‘QFKDNVILL’, HLA_allele)

#### Data analysis.

A variety of postprocessing options for data analysis can be incorporated into a workflow. These include binding mode rescoring or peptide ranking with DINC and structure visualization with NGL Viewer,^13^ among others. Minimal example: arena.visualize(structure)

For a smooth user experience, all computational tools involved in HLA-Arena are packaged within a Docker image (Appendix provides installation details), thus eliminating the burden of managing software dependencies. Another advantage of Docker containerization is that it makes HLA-Arena platform agnostic. As a result, it can be deployed on a desktop computer or a high-performance computing cluster, across different operating systems. Users can customize available workflows by adding modeling or analysis steps. We plan to continuously expand the capabilities of HLA-Arena by providing support for additional tools.^29-31^

## RESULTS

We now present the results we obtained when carrying out 3 different workflows that exemplify the diversity of applications offered by HLA-Arena. Each workflow leverages the functionalities of several tools in a coherent scenario.

### Geometry Prediction of pHLA Binding Modes

HLA-Arena can be used to predict conformations of peptides bound to HLA receptors, even for peptides presenting unusual binding modes.^10^ To illustrate this, using the geometry prediction workflow based on DINC (Fig 3), we tried to reproduce the crystal structures of 2 such peptides.

**FIG A1.**
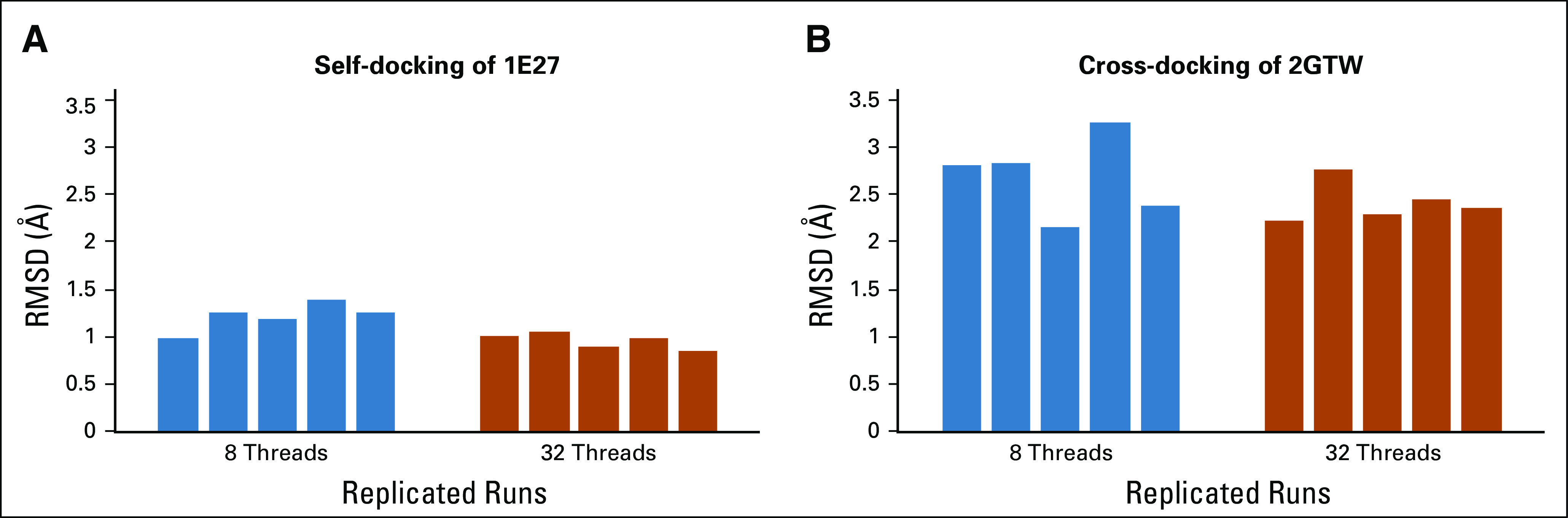
Lowest root mean square deviation (RMSD) binding modes sampled by DINC in the geometry prediction workflow. (A) Results of a self-docking experiment aimed at reproducing a crystal structure (with Protein Data Bank [PDB] code 1E27) involving a 9-mer peptide derived from HIV-1 and the HLA-B*51:01 receptor. This experiment was carried out with either 8 or 32 threads. Each bar corresponds to the so-called top RMSD conformation (ie, the conformation with the lowest RMSD to the target crystal structure) sampled in each of 5 replicated runs. Near-native peptide conformations (ie, conformations with an all-heavy-atom RMSD to the crystal structure < 2.5 Å) were sampled in all runs. The best conformation across all runs had an all-heavy-atom RMSD of 0.84 Å. (B) Results of a cross-docking experiment aimed at reproducing a crystal structure (with PDB code 2GTW) involving HLA-A*02:01 and a 9-mer peptide derived from the MART-1/melan-A protein. Near-native peptide conformations were sampled in 2 of 5 runs when using 8 threads and in 4 of 5 runs when using 32 threads. The best conformation sampled across all runs had an all-heavy-atom RMSD of 2.15 Å.

First, we conducted a self-docking experiment with a crystal structure (with PDB code 1E27) involving HLA-B*51:01 and a 9-mer peptide derived from HIV-1. It has been suggested that the fifth residue acts as a secondary anchor for this peptide, leading to structural rearrangement of its central and amino-terminal residues.^32^ Our experiment evaluated the capability of DINC to reproduce the bound geometry of this peptide, without considering receptor flexibility. To evaluate performance and reproducibility, we carried out this experiment with either 8 or 32 threads (for the parallel process in DINC), running 5 replicates in each case. Default values were used for other DINC parameters.^23^ Results (Appendix Fig A1A) show that, in every single run, HLA-Arena sampled a near-native peptide conformation (ie, a conformation with an all-heavy-atom root mean square deviation [RMSD] to the crystal structure < 2.5 Å).

**FIG 4.**
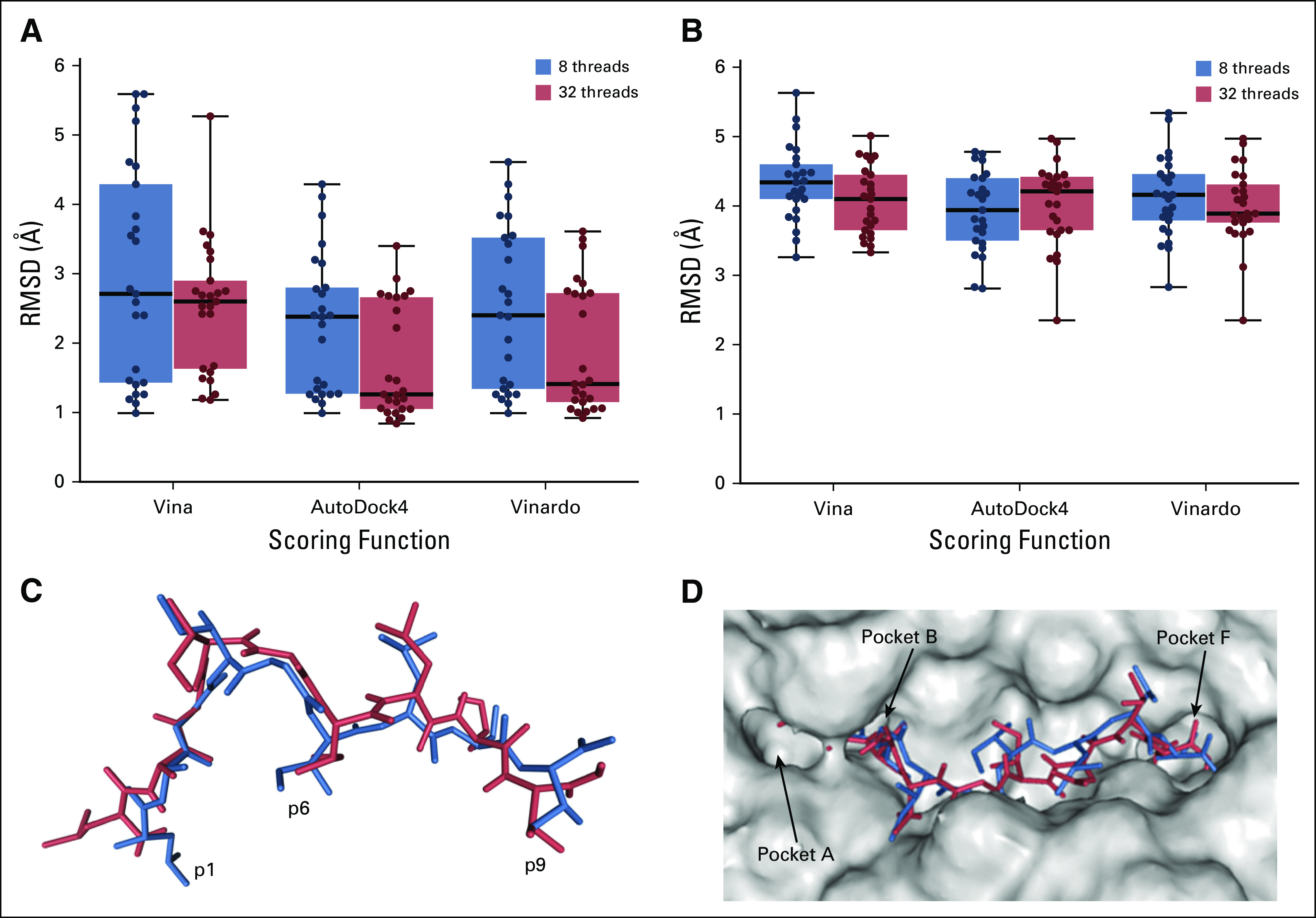
Geometry prediction of peptide-HLA binding modes. (A) Three scoring functions are used to select the top-5 ranking conformations produced by 5 replicates of a self-docking experiment aimed at predicting the binding mode of a 9-mer peptide (under Protein Data Bank [PDB] code 1E27) using 8 or 32 threads for DINC. Each box plot aggregates results of the 5 replicates. Each dot corresponds to a conformation plotted according to its all-heavy-atom root mean square deviation (RMSD) to the reference crystal structure. (B) Results of a cross-docking experiment aimed at predicting the binding mode of a 9-mer peptide (under PDB code 2GTW) obtained with the same methodology. (C) Side view of the best binding mode (red) identified by AutoDock4 and Vinardo and aligned with the crystal structure (blue) of this peptide (under PDB code 2GTW). Only heavy atoms are depicted, using a sticks representation. Note that this sampled conformation has an all-heavy-atom RMSD of 2.35 Å and does not perfectly reproduce the side-chain arrangement of the first residue. A better conformation, with an all-heavy-atom RMSD of 2.15 Å, was sampled by HLA-Arena (Appendix Fig A2) but was not among the top-ranking conformations. (D) Top view of the HLA binding site (depicted by a gray surface) with peptide conformations shown in panel C within it (as sticks). This peptide uses its first amino acid as primary anchor (ie, residue p1 is anchored in pocket B), which is quite unusual for HLA-A*02:01 binders. Images in panels C and D were generated with HLA-Arena using the embedded NGL Viewer.^13^ Both images were edited to add labels.

Geometry prediction involves 2 issues that are especially challenging with peptides.^5^ The first relates to sampling (ie, how to explore the full flexibility of a large ligand). The second relates to scoring (ie, how to identify the best ligand conformation in a pool of diverse binding modes). HLA-Arena relies on the incremental process of DINC to overcome the sampling issue. It also includes a filtering step to remove peptide conformations with reverse orientation in the binding cleft. To address the scoring issue, HLA-Arena makes use of multiple scoring functions. For instance, in this self-docking experiment, conformations were ranked with the scoring functions of AutoDock4,^33^ Vina,^20^ and Vinardo.^34^ All 3 scoring functions were able to identify near-native conformations. However, in the case of AutoDock4 (Fig 4A), the top-5 ranking conformations in 1 of the replicates included the overall lowest RMSD conformation (ie, the conformation with the lowest RMSD to the crystal structure among all sampled conformations; Appendix Fig A1A).

**FIG A2.**
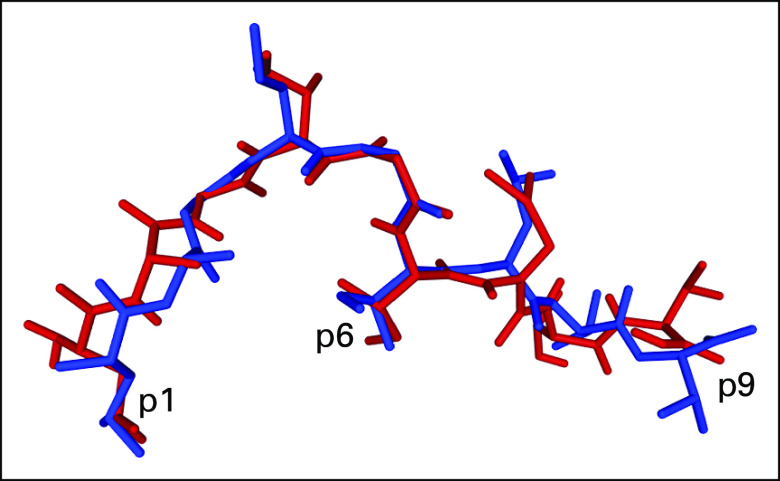
Lowest root mean square deviation (RMSD) binding mode sampled in a cross-docking experiment. Depicted in red is the lowest RMSD conformation sampled by DINC in the cross-docking experiment aimed at reproducing a crystal structure (with Protein Data Bank code 2GTW) involving HLA-A*02:01 and a 9-mer peptide derived from the MART-1/melan-A protein. The all-heavy-atom RMSD of this conformation to the crystal structure (depicted in blue) is only 2.15 Å. This conformation accurately reproduces the geometry of the first residue (p1), which has an unusual arrangement (ie, anchored in pocket B of the binding cleft).

Second, we tried to reproduce a crystal structure (with PDB code 2GTW) involving HLA-A*02:01 and a 9-mer peptide derived from the MART-1/melan-A protein.^35^ This peptide has an A27L substitution in comparison with the MART-1 peptide targeted by numerous clinical studies.^36,37^ This substitution leads to an alternative arrangement of primary anchor residues, resulting in an unusual binding mode.^10,30,35^ Again, we ran 5 replicates of the geometry prediction workflow, using either 8 or 32 threads. For the prediction task to be closer to a real-case scenario, we performed a cross-docking experiment, accounting for receptor flexibility. It made this task much harder, from both sampling and scoring perspectives.^38,39^ Despite this, HLA-Arena sampled near-native conformations, although it performed better when using 32 threads (Appendix Figs A1B and A2). In terms of scoring, only AutoDock4 and Vinardo were able to recover near-native conformations (Fig 4B). Note that HLA-Arena also allows visualization of the 3-dimensional structure of the top-ranking binding mode (Figs 4C and 4D).

### Structure-Based Prediction of Binding Energy

**FIG 5.**
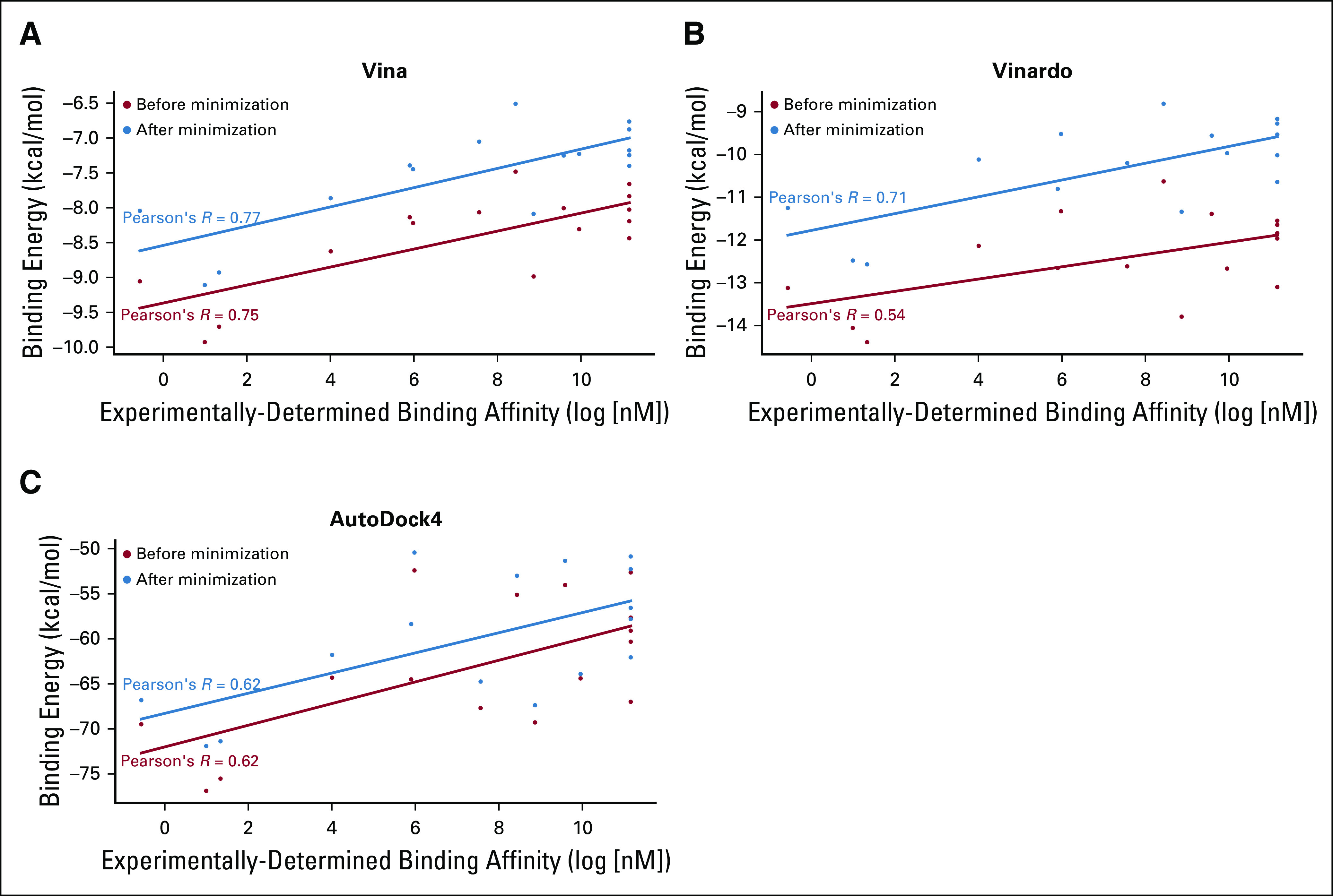
HLA-A*02:01 binding predicted for a small set of peptides. Each plot illustrates the correlation between experimentally obtained binding affinities (extracted from the Immune Epitope Database) and structure-based binding energies, as predicted by a given scoring function: (A) Vina, (B) Vinardo, and (C) AutoDock4. Structures were generated with APE-Gen, with or without minimization with OpenMM. Correlation coefficients are also reported (as Pearson’s *R*). Each point corresponds to a peptide in Appendix Table A1.

To demonstrate another application of HLA-Arena, we used the binding prediction workflow (Fig 3) to predict binding to HLA-A*02:01 for a small data set of selected peptides (Appendix Table A1). This data set included 5 experimentally identified nonbinders, as well as 11 binders with experimental binding affinities available in the Immune Epitope Database^40^ and crystal structures in complex with HLA-A*02:01 available in the PDB. For each peptide, we generated an ensemble of bound conformations with APE-Gen. The binding energy of each peptide was then estimated as the median score within the conformation ensemble for each scoring function (ie, AutoDock4, Vina, and Vinardo). Correlations between these predicted binding energies and experimentally determined binding affinities were then determined (Fig 5).

In addition to the default local optimization performed by APE-Gen, HLA-Arena provides the option of minimizing the resulting complexes with OpenMM.^24^ To evaluate the impact of this procedure, we recalculated binding energies and correlations after running this energy minimization for all conformations in each ensemble. Our results showed a consistent increase of the predicted binding energies for all scoring functions (Fig 5). This might reflect the differences in binding energy estimation that exist between these empirical or semi-empirical scoring functions^41^ and the force field used by OpenMM (ie, amber99sbildn).^24^ Despite increasing binding energies, the OpenMM minimization had a positive impact on overall correlations.

Interestingly, the best correlation with experimental binding affinities was obtained when using Vina. This result is in agreement with previous studies evaluating the performance of Vina in virtual screening of drug-like ligands.^41,42^ Note that contrary to the geometry prediction workflow, in which a scoring function was only used to rank different conformations of a given peptide, here the scoring function also had to rank different peptides. Although the same function can be used for both purposes,^5^ it is possible that better results are obtained when using functions optimized for each task.

**FIG A3.**
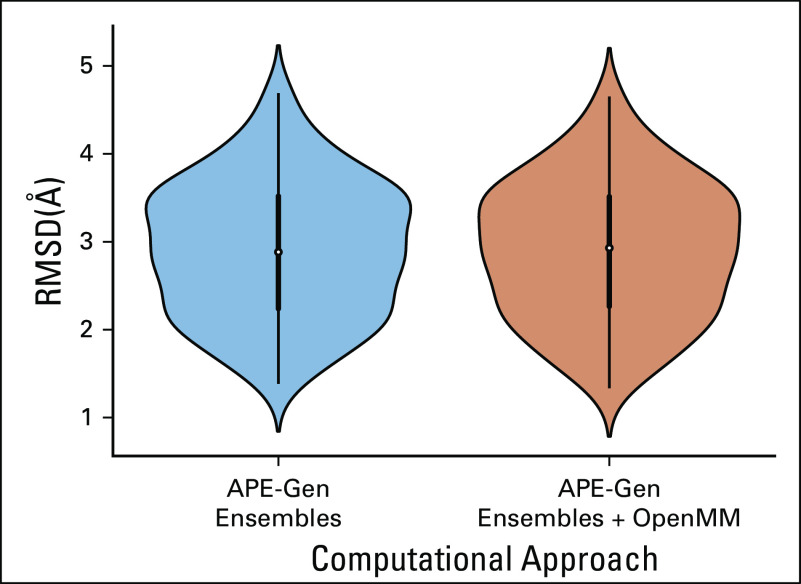
Binding mode ensembles generated by APE-Gen include near-native peptide conformations. This plot aggregates the all-heavy-atom root mean square deviation (RMSD) between each conformation produced by APE-Gen for each peptide binder in our data set (Appendix Table A1) and its reference crystal structure. Results for conformations having undergone energy minimization with OpenMM^24^ are also reported, although differences are subtle. These conformations were produced by a single round of sampling with APE-Gen.

**FIG A4.**
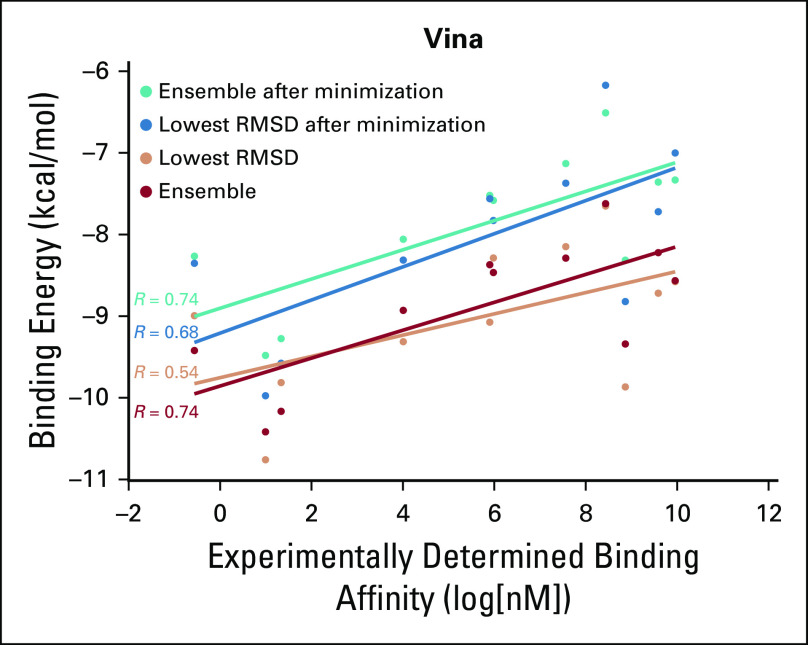
Binding energy rankings associated with ensembles or single conformations. This plot reports correlations (assessed as Pearson’s *R*) between experimentally determined binding affinities and structure-based binding energies predicted by the Vina scoring function using different procedures. More specifically, the binding energy of a given peptide can be defined as: the score of the conformation with the lowest RMSD to the crystal structure in the ensemble produced by APE-Gen (*R* = 0.54), the score of that same conformation minimized with OpenMM (*R* = 0.68), the median score within the ensemble of conformations produced by APE-Gen (*R* = 0.74), or the median score within that same ensemble after minimization with OpenMM (*R* = 0.74). Each point corresponds to a known peptide binder to HLA-A*02:01 (Appendix Table A1). Note that the nonbinders were not included in this analysis.

For the HLA-A*02:01 binders in our data set, we can compute RMSDs between their associated crystal structures and conformations generated by APE-Gen. This allows verification that APE-Gen ensembles include near-native conformations (Appendix Fig A3) and evaluation of the impact of the OpenMM minimization on these conformations. This also allows comparing the use of an ensemble of conformations to predict binding energies with the use of a single conformation from this ensemble (eg, the conformation with the lowest RMSD to the corresponding crystal structure). Our results with the Vina scoring function suggest that better correlations are obtained with ensembles of conformations (Appendix Fig A4).

### Virtual Screening of Tumor-Derived Peptides

HLA-Arena allows researchers to perform, for the first time to our knowledge, a large-scale structure-based virtual screening of HLA-binding peptides. In addition, by combining sequence- and structure-based methods, HLA-Arena represents a fresh alternative for the identification of tumor-derived peptide targets considering patient-specific HLAs. To demonstrate this application, we used the virtual screening workflow (Fig 3) to predict which peptides were the strongest binders to the class I HLA receptors of a fictitious patient with cancer.

We considered 6 alleles: HLA-A*24:02, HLA-A*26:01, HLA-B*15:01, HLA-B*35:01, HLA-C*04:01, and HLA-C*05:01. We built a peptide data set by selecting 500 known binders and 1,000 decoys for each allele, for a total of 9,000 peptides. Sequences of known binders were obtained from the SysteMHC Atlas,^43^ where they were derived from immunopeptidomics studies. Sequences of decoys were obtained from the training set of NetMHCpan.^44^

First, the whole data set of peptides was screened for HLA binding with MHCflurry,^12^ using an affinity threshold specified by the user. This allows the user to quickly select the most likely binders for each HLA receptor, before moving on to the more computationally expensive steps. In this example, a threshold of 500 nM selected 2,604 peptides. Then, we proceeded with the structural modeling of the full pHLA complex for all selected peptides. Finally, peptides were ranked based on binding energies derived from the modeled structures. The entire pipeline took 86 hours on a desktop computer or 5 hours on a high-performance cluster (Appendix).

**FIG 6.**
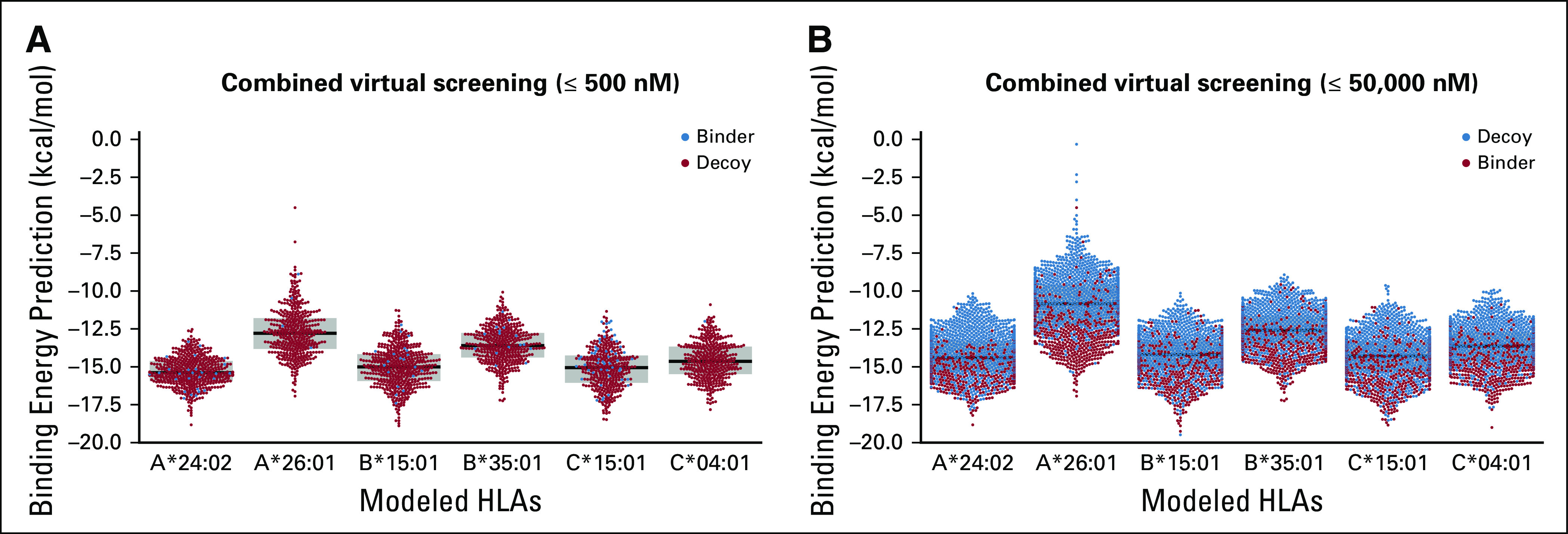
Structure-based virtual screening for high-affinity HLA binders. The HLA-Arena virtual screening workflow was used to predict peptide binders for 6 HLA receptors of interest. For this exercise, a data set of 9,000 peptides was created, using 500 known binders (red dots) and 1,000 decoys (blue dots) for each HLA. (A) Results of a combined virtual screening (ie, MHCflurry plus APE-Gen) with a 500-nM threshold for MHCflurry. (B) Results of the same virtual screening using a 50,000-nM threshold for MHCflurry. In both plots, each dot corresponds to the top-scoring conformation of a modeled peptide-HLA complex, selected from the ensemble of conformations produced by APE-Gen. For each HLA (on the *x*-axis), complexes with the lowest-binding energies (on the *y*-axis) would be predicted as the best candidates for further analysis or experimental validation.

The threshold used in MHCflurry directly affects the sensitivity/specificity of the overall prediction. Recent surveys indicate that commonly used thresholds for sequence-based HLA binding predictors (eg, 500 nM) can yield a sensitivity as low as 40%,^45^ with great variation in accuracy between HLA alleles.^46^ On our data set, a 500-nM threshold produced several false-positive (Fig 6A, blue dots) and false-negative predictions (data not shown). In trying to address this issue, we observed that our structure-based analysis could usually eliminate at least half of the false-positive predictions and recover significant numbers of false-negative predictions, although results varied depending on the studied HLA allele (data not shown).

Because our workflow allowed variation of the MHCflurry threshold, we repeated the aforementioned virtual screening experiment with a 50,000-nM value. This led to all 9,000 peptides being selected for modeling and ranking. The observed enrichment of true binders among the top-ranking peptides (Fig 6B, red dots at the bottom of the distributions) further corroborates our claim that structural information is useful when screening HLA binders.

In these examples, we performed only 1 sampling round in APE-Gen for each complex, and only the top-scored conformation was used as input for ranking. Better results could be obtained by executing more sampling rounds in APE-Gen, performing the OpenMM minimization, or using the whole APE-Gen ensemble. More importantly, accurate scoring remains an open challenge. Therefore, structure-based predictions cannot yet outperform sequence-based methods, but they can be combined to provide additional information when selecting peptides for experimental validation.

## DISCUSSION

HLA-Arena provides researchers with a customizable environment to create and execute sophisticated workflows for the structural modeling and analysis of pHLA complexes. Its intuitive interface relies on Jupyter Notebook and Docker to dramatically reduce the burden of software dependencies and the need for advanced programming skills, making its resources accessible to a wide audience. Available workflows combine commonly used software for protein modeling and analysis, with tools that we developed to address challenges specific to pHLA complexes. We believe that HLA-Arena could become a stepping stone toward a broad collaborative effort to study pHLA complexes.

In this report, we present 3 workflows to showcase the capabilities of HLA-Arena. First, HLA-Arena enabled the geometry prediction of pHLA structures, even for peptides with unusual binding modes, by using template-free molecular docking. Second, HLA-Arena allowed prediction of binding energies for potential HLA binders by quickly producing ensembles of bound conformations for these peptides and rescoring all the results. Third, HLA-Arena enabled a more accurate virtual screening of HLA binders by combining sequence- and structure-based approaches.

These workflows can be modified to allow for additional analysis of the modeled pHLA complexes (eg, to perform molecular dynamics with OpenMM^47,48^ or cross-reactivity assessment).^2,49,50^ Thanks to high-performance computing and efficient sampling, molecular dynamics could play a bigger role in providing accurate estimates of pHLA binding affinity and complex stability.^51,52^

HLA-Arena can be integrated into computational pipelines for basic cancer research or to help inform physicians in preclinical settings. It can be used to perform the large-scale modeling and selection of tumor-associated peptides, computer-aided design of altered peptide ligands, and study of T-cell cross-reactivity.^2,8^ In addition to HLA binding prediction, immunotherapy applications require identification of peptides that are uniquely displayed by cancer cells. This important task will be addressed in future updates of HLA-Arena.

It is important to note that HLA-Arena provides efficient solutions to sampling challenges associated with pHLA modeling^8,23^ and facilitates the integration of these solutions with other tools for structural analysis. However, the accuracy of structure-based peptide ranking is limited by existing scoring functions. As they improve, new scoring functions will be incorporated into HLA-Arena to replace current ones or be combined with consensus methods.^53,54^ In time, we expect that structure-based analyses will become essential to peptide target prediction for neoantigen discovery, vaccine development, and cancer immunotherapy, especially for patients with less prevalent HLA alleles.

## Figures and Tables

**TABLE A1.**
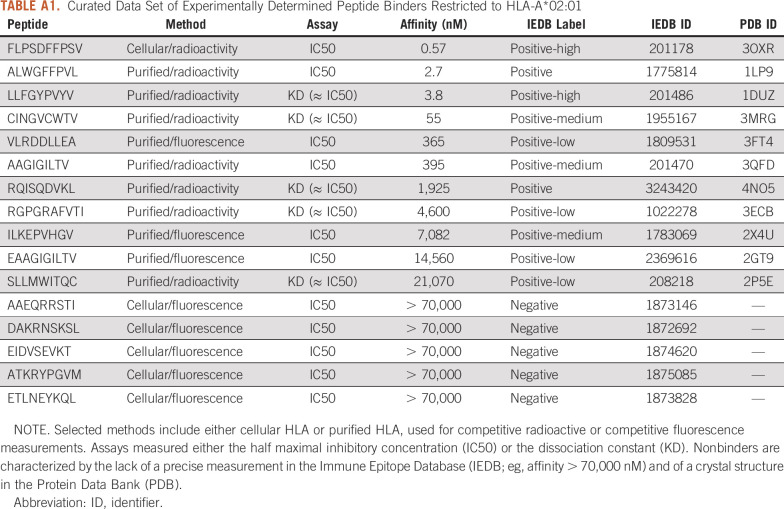
Curated Data Set of Experimentally Determined Peptide Binders Restricted to HLA-A*02:01

## APE-Gen: Fast Generation of pHLA Binding Mode Ensembles

We recently released a new tool, the anchored peptide-HLA (pHLA) ensemble generator (APE-Gen), which produces an ensemble of binding modes for a pHLA complex, starting from the sequences of a peptide and HLA receptor.^8^ APE-Gen involves an iterative process repeating the 3 following steps. First, the ends of the peptide backbone are anchored within known pockets in the HLA binding site using available backbone termini templates. Second, the peptide backbone is completed by applying the random coordinate descent loop modeling tool,^19^ which efficiently yields several valid backbone conformations. Third, side chains are added to the backbone conformations, and local optimization is performed with Smina^25^ to fix steric clashes. This step considers full-peptide flexibility and binding site side-chain flexibility, producing a set of full-atom peptide conformations within the HLA binding site. After each such round of sampling, the highest-quality conformation (according to the internal scoring function, currently Vinardo) can be used as a template for the next round.

By generating a diverse ensemble of pHLA binding modes, APE-Gen implicitly accounts for the natural flexibility of peptides within the binding site. We showed that APE-Gen could reproduce the entire set of nonredundant classic class I pHLA structures available in the Protein Data Bank^27^ (535 complexes at the time of the study).^8^ In that case, we used a single round of sampling per complex. The average root mean square deviation (RMSD) between modeled peptides and their corresponding crystal structure (considering all heavy atoms) was only 2.02 Å, which is considered an accurate reproduction. Even better results can be obtained when performing optimization and/or additional rounds of sampling, especially for longer peptides.^8^

APE-Gen is computationally efficient, producing dozens of binding modes in a few minutes on a standard desktop computer. It can be run for several peptides and a given HLA receptor, thus producing valuable information for peptide ranking and binding affinity prediction and enabling structure-based virtual screening of HLA binding peptides. We have also shown the potential benefits of APE-Gen when studying T-cell cross-reactivity.^8^

## DINC: Incremental Docking of pHLA Complexes

In previous work, we presented a molecular docking approach called DINC (which stands for docking incrementally), specifically developed for large ligands, including peptides.^21^ The underlying idea is to incrementally dock larger and larger fragments of a ligand, instead of trying to dock it all at once. Note that this incremental docking process focuses on ligand flexibility, although selected receptor side chains can also be sampled. This process is parallelized to allow for broader sampling, by having several runs of docking performed independently at each step and grouping their results together. DINC is also a meta-docking method, in the sense that it relies on existing molecular docking tools, such as AutoDock4,^33^ Vina,^20^ and Smina^25^ to perform the docking of the fragments at each step. As a consequence, fragment sampling and scoring can be performed by different tools.

The latest version of our software, called DINC 2.0, has been made available as a Web server.^9^ We recently showed that it performs a more exhaustive sampling than other docking approaches.^23^ In that study, DINC was benchmarked using 5 public data sets including large ligands; it reproduced many crystal structures on which other docking tools had failed.^23^ For example, it has been used to study the inhibition of the Src homology 2 domain of STAT3 by peptidomimetics.^22^ We also showed that DINC could reproduce a diverse set of pHLA structures encompassing 10 HLA alleles and peptides with diverse binding modes; it achieved an average all-heavy-atom RMSD of 1.92 Å.^10^ Note that DINC is not limited to common class I HLA receptors, contrary to many related tools.^5^ It can be applied to complexes involving synthetic ligands, to rare and nonclassical class I HLAs, and potentially to class II HLA receptors.^9^ An updated version of DINC is made available through Docker Hub (docker pull kavrakilab/dinc-bin).

## HLA-Arena Performance for Virtual Screening

HLA-Arena provides the most efficient workflow available for structure-based virtual screening of HLA binders. For the experiment we report in the Results section, the breakdown of computing time is as follows: MHCflurry^12^ needs approximately 15 seconds to screen the entire data set of 9,000 peptides. The homology modeling step takes approximately 3 minutes for each HLA allele and can be skipped for HLAs with available crystal structures. The APE-Gen step takes approximately 2 minutes per pHLA complex on a desktop computer with 6 to 8 threads. The (optional) rescoring takes approximately 2 seconds per complex using an HLA-Arena function that relies on Smina.^25^ Therefore, running the entire workflow on a desktop computer takes approximately 86 hours with an MHCflurry threshold at 500 nM and approximately 300 hours with an MHCflurry threshold at 50,000 nM. This running time can be dramatically reduced if the APE-Gen step is executed on a cluster. For instance, on a machine with 64 threads, with an MHCflurry threshold at 500 nM or 50,000 nM, the same workflow could be executed in 5 or 19 hours, respectively (without rescoring). Future updates of HLA-Arena should provide additional resources for running workflows in a remote high-performance computing cluster.

## HLA-Arena Installation

1. If you do not already have it, install Docker for Mac or Windows (https://www.docker.com/products/docker-desktop) or for Linux (https://docs.docker.com/install).

2. In a command prompt, pull the HLA-Arena image from Docker Hub by typing: docker pull kavrakilab/hla-arena

3. Create a folder in which you want to run the workflows (optional).

4. Copy HLA-Arena notebooks and associated data to your local machine by typing: docker run --rm -v $(pwd):/temp--entrypoint cp kavrakilab/hla-arena /hla_arena_data/data.tar.gz \ /temp/; tar -xzvf data.tar.gz

5. Run HLA-Arena in this folder by typing: docker run --rm -v $(pwd):/data -p 8888:8888 \ --entrypoint=“” kavrakilab/hla-arena jupyter \ notebook --port=8888 --no-browser \ --ip=0.0.0.0 --allow-root.

6. This should generate a URL with the following format: http://127.0.0.1:8888/?token=<token_value>.

7. Copy and paste this URL into a browser, and open any available Jupyter notebook (ie, 1 of the files with extension .ipynb). Note that all the data created in the container will be saved inside subdirectories of the current folder.

8. Check out the file DOCUMENTATION (https://kavrakilab.github.io/hla-arena/DOCUMENTATION.html) for additional information on the workflows and available functions.
